# Variation in Actinobacterial Community Composition and Potential Function in Different Soil Ecosystems Belonging to the Arid Heihe River Basin of Northwest China

**DOI:** 10.3389/fmicb.2019.02209

**Published:** 2019-09-24

**Authors:** Binglin Zhang, Xiukun Wu, Xisheng Tai, Likun Sun, Minghui Wu, Wei Zhang, Ximing Chen, Gaosen Zhang, Tuo Chen, Guangxiu Liu, Paul Dyson

**Affiliations:** ^1^State Key Laboratory of Cryospheric Sciences, Northwest Institute of Eco-Environment and Resources, Chinese Academy of Sciences, Lanzhou, China; ^2^Key Laboratory of Extreme Environmental Microbial Resources and Engineering, Lanzhou, China; ^3^Key Laboratory of Desert and Desertification, Northwest Institute of Eco-Environment and Resources, Chinese Academy of Sciences, Lanzhou, China; ^4^College of Geography and Environmental Engineering, Lanzhou City University, Lanzhou, China; ^5^College of Animal Science, Gansu Agricultural University, Lanzhou, China; ^6^Institute of Life Science, Swansea University Medical School, Swansea, United Kingdom

**Keywords:** actinobacterial community, diversity, vegetation gradient, arid region, Heihe river

## Abstract

Actinobacteria are known for their metabolic potential of producing diverse secondary metabolites such as antibiotics. Actinobacteria also playimportant roles in biogeochemical cycling and how soils develop. However, little is known about the effect of the vegetation type on the actinobacterial community structures in soils from arid regions. For these reasons, we have analyzed the actinobacterial communities of five types of ecosystem (tree grove, shrub, meadow, desert, and farm) in the Heihe river basin. Using 16S rRNA high-throughput sequencing, we found 11 classes of Actinobacteria, with dominant classes of Actinobacteria (36.2%), Thermoleophilia (28.3%), Acidimicrobiia (19.4%). Five classes, 15 orders, 20 families and 36 genera were present in all samples. The dominant generalist genera were *Gaiella*, *Solirubrobacter*, *Nocardioides*, *Mycobacterium*, and *Pseudonocardia*. The actinobacterial community structures were significantly affected by the environment and vegetation type. The diversity of the actinobacterial community in the desert ecosystem was high, and this ecosystem harbored the highest proportion of unclassified sequences, representing rare Actinobacteria. Functional metagenomic prediction, using PICRUSt, indicated that Actinobacteria play an important role in nitrogen cycling in both desert and cultivated farm ecosystems.

## Introduction

The phylum Actinobacteria is a primary eubacterial phylogenetic clade containing diverse gram-positive bacteria (Sun et al., 2010) that belong to several classes such as the Acidimicrobiia, Actinobacteria, and Thermoleophila. Most Actinobacteria are aerobic saprophytes, and many can produce bioactive compounds (Eisenlord and Zak, 2010; Miao and Davies, 2010). Many of these secondary metabolites are used in medicine and agriculture, including antibacterial, antifungal and antitumor drugs (Hill et al., 2011). Unexplored and underexplored niche habitats are important resources of rare actinomycetes, and consequently potential new bioactive compounds. The distribution of different Actinobacteria is associated with environmental factors, such as soil type and pH (Hayakawa et al., 1988; Matsukawa et al., 2007; Hayakawa, 2008).

Actinobacteria are typically dominant soil microbes (Hill et al., 2011). They are important for the cycling of carbon, nitrogen, phosphorus, potassium, and several other elements in the soil (Goodfellow and Williams, 1983; Holmalahti et al., 1994; Hill et al., 2011). As saprophytes, they produce a range of extracellular hydrolytic enzymes which can degrade animal and plant polymers, including lignin, cellulose, chitin, and other organic compounds (Eisenlord and Zak, 2010). The actinomycete genus *Frankia* are root symbionts incorporated into nodules of a wide range of perennial woody dicotyledonous plants (Normand et al., 1996). However, in many cases the ecological roles of Actinobacteria in soils are poorly understood (Miao and Davies, 2010). A better understanding of the diversity and distribution of Actinobacteria can provide insight into microbial ecology and guide the discovery of novel bioactivities such as new antibiotics (Beattie et al., 2011; Hill et al., 2011).

The diversity of Actinobacteria has been investigated in several special or extreme environments such as marine sediments (Zhang et al., 2014; Duran et al., 2015), volcanic caves (Riquelme et al., 2015), sediments of cold springs (Yang et al., 2015), microbial mats of hot springs (Jiang et al., 2012), glacial forelands (Zhang et al., 2016b), lakes (Parveen et al., 2011), associations with organisms (Inthasotti and Pathom-aree, 2015), and deserts (Ding et al., 2013). In this study, we have investigated the Heihe River basin located in the arid northwest region of China. It offers both a special environment and an untapped resource of potential new actinobacterial diversity. Previous research into microbial diversity in the Heihe River has been limited to studies that associate watershed quality with bacterial community structures in the middle reach of the river basin (Borruso et al., 2015). In addition, Su et al. (2004) investigated bacterial diversity in the lower reach of the river using culture-dependent methods. Moreover, Tai et al. (2013) studied the diversity of nitrogen-fixing bacteria in the upper reaches of the Heihe River.

Habitat types can significantly affect soil bacterial diversity (Han et al., 2007; Xu et al., 2014). The Heihe River basin is an ideal place to research microbial diversity in relation to the environment. The vegetation types of the region vary from low to high altitudes (Wang et al., 2009; Tai et al., 2013). The abiotic (e.g., soil) and biotic (e.g., vegetation) varied with slope aspect (Qin et al., 2016). Soil organic carbon density ranged from 9.73 to 35.21 kg m^–2^ at 0–60 cm at the hill scale (Zhu et al., 2017). Measures of actinobacterial diversity in relation to vegetation can be expected to provide insights into the ecological role of the bacteria. Insight into soil ecology can be gained from understanding how microbial communities influence functional diversity in soil (Xu et al., 2014). However, studies into community composition based on16S rRNA gene sequences have generally neglected the functional and metabolic properties of a microbial community (Langille et al., 2013). The aims of this study were to: (1) investigate actinobacterial communities in soils of the Heihe River basin with samples taken from tree groves, shrubland, meadows, desert and farmland, (2) assess any relation of the vegetation gradient (tree groves, shrubland, meadow, desert) to the actinobacterial communities, and (3) assess to what extent metabolic pathways differ among the Actinobacteria found in the soils of these different habitats.

## Materials and Methods

### Site Description and Soil Sampling

The Heihe River basin in northwestern China represents the second-largest inland river basin in this arid region (37°41′-42°42′N and 96°42′-102°00′E) (Wang et al., 2009). The Heihe River basin extends from its upper reaches located on the northern slopes of the Qilian Mountains (Li et al., 2001). Precipitation mainly takes place in the summer months and decreases from east to west while increasing from approximately 200 mm at low altitudes, to 600 mm at high altitudes. The types of environments found consist of the following zones: desert steppe, arid shrubbery grassland, forest grassland, sub-alpine shrubbery meadow, alpine cold-and-desert meadow, and alpine perma-frost-snow-ice from low to high altitudes, respectively (Wang et al., 2009; Tai et al., 2013). The lower reaches of the Heihe River basin are located on the northern Alxa Highland. It is one of the most arid regions in China. The precipitation is concentrated during the summer. Annual mean precipitation is 42 mm, with an annual mean evaporation of 3755 mm (Li et al., 2001; Yin et al., 2012).

Soil samples were collected from five types of natural soil environments tree groves, shrubland, meadow, desert, and farmland throughout July 27th to Aug 14th, 2012 (Table 1 and Supplementary Figure S1). For tree grove, shrub and meadow ecosystems, four different representative plant species typical to each habitat were chosen for soil sampling. In the desert, we chose soils representing four different degrees of desertification. For farmland, soil samples treated with four different fertilizers were chosen. More detailed information about sample sites is provided in Table 1. The desert soil samples were obtained exclusively from the lower reaches of the Heihe River basin. In each sample site, we collected three independent replicate samples and each replicate was pooled from five sample soils (four corner and one centre sample) that were 5 cm deep within an area of 2 m^2^. For each sample, approximately 10 g of soil was obtained with a trowel and sieved to remove non-particulate content. Samples from shrub and tree groves were obtained from soil adjacent to the relevant non-mixed plant species. The samples were kept below 4°C during transport to the laboratory and were then stored at −20°C until DNA extraction.

**TABLE 1:** Overview of the soil sample locations.

**Biotopes**	**Vegetation/**	**Abbr.**	**Latitude**	**Longitude**	**Elevation**
	**Soil Type**				
Tree grove soil	*Sabina przewalskii*	T1	38.0706	100.2402	3479
	*Picea crassifolia*	T2	38.1271	100.1485	3094
	*Salix babylonica*	T3	38.1742	100.2164	2820
	*Populus tremula*	T4	38.1736	100.2138	2814
Shrubland soil	*Caragana jubata*	S1	38.0300	100.2333	3894
	*Potentilla fruticosa*	S2	38.0652	100.2146	3624
	*Hippophae rhamnoides*	S3	38.0642	100.2656	3250
	*Salix wilhelmsiana*	S4	38.0681	100.2964	3075
Meadowland soil	*Carex tristachya*	M1	38.1216	100.1914	3128
	*Stipa capillata*	M2	38.2508	100.2181	2911
	*Kobresia pygmaea*	M3	38.1769	100.2150	2905
	*Achnatherum splendens*	M4	38.2593	100.1809	2631
Desert soil	Mild desertification	D1	42.0203	101.2385	922
	Moderate desertification	D2	42.0179	101.2587	915
	Severe desertification	D3	42.0211	101.2618	933
	Extreme desertification	D4	42.0328	101.2952	916
Farmland soil	M fertilizer	F1	38.3518	100.1319	1380
	N/P/M fertilizer	F2	38.3532	100.1344	1380
	N/P/K fertilizer	F3	38.3515	100.1323	1380
	N/P/K/M fertilizer	F4	38.3529	100.1338	1380

### DNA Extraction and Sequencing

DNA was isolated from representative 0.5 g samples of the triplicate mixed soils or from 2 g samples of desert soil, using a PowerSoil DNA Isolation Kit (MoBio Mo Bio Laboratories, Inc., Carlsbad, CA). We amplified the V4 region of the bacterial 16S rRNA genes with the bacterial universal primers 515F and 806R. The PCR amplification conditions were set exactly as described previously (Zhang et al., 2016a). The amplicons were sequenced with an Illumina platform.

### Sequence Analyses

Raw sequence reads were sorted with barcodes and quality trimmed using QIIME 1.91 (Caporaso et al., 2010) in Bio-Linux 8.0.5 (Field et al., 2006). The following parameters were used: minimum 200 bp in length, with perfect matches to the sequence tag (barcode) and the 16S rRNA gene primer, and exclusion of sequences with undetermined bases, including chimeric sequences. Remaining sequences were clustered into operational taxonomic units (OTUs) with a minimum of 97% identity, using usearch v11.0 (Edgar, 2010) on QIIME followed by taxonomically assigning each sequence with RDP (Wang et al., 2007) according to the best matches in the SILVA ribosomal database (version 132) (Quast et al., 2013; Yilmaz et al., 2014). Sequences belonging to the phylum Actinobacteria were identified with QIIME from each library (filter_taxa_from_otu_table.py, –positive_tax p__Actinobacteria). Specialists were defined as either families or genera that were present in a maximum of two out of 20 samples, whereas generalists were defined as families of genera present in more than 18 samples. Occupancy indicates from how many of the sites a family or genus was recovered. Sequences obtained from this research were deposited at DDBJ/EMBL/GenBank with accession number KCVG00000000.

### Metagenome Analyses

We used PICRUSt to predict actinobacterial community functional content based on 16S rRNA sequences (Langille et al., 2013). To identify operational taxonomic units (OTU) within each ecosystem, the 16S rRNA sequences were compared with sequences in the Greengenes ribosomal database (version 13-5). A minimum of 97% identity was used to classify each OTU using pick_closed_reference_otus.py in QIIME (DeSantis et al., 2006). The data for each ecosystem was normalized in terms of the numbers of sequences representing each OTU, using normalize_by_copy_number.py in PICRUSt. Subsequently, the script predict_metagenomes.py in PICRUSt was used to predict metagenome function for a given OTU, with reference to the Kyoto Encyclopedia of Genes and Genomes database (Kanehisa and Goto, 2000). A full description of the methods is detailed in Cleary et al. (2015). The contributions of genes and selected orders for each sample were analyzed with the metagenome contributions.py script in PICRUSt.

### Statistical Analyses

The alpha diversity of samples was calculated with alpha_diversity.py in QIIME with the lowest number of sequences of all samples. The dissimilarity of the actinobacterial communities and metagenome gene functional content were determined using beta_diversity.py in QIIME following the Bray-Curtis method (beta_diversity.py -i otu_table.biom -o otu_beta_diversity -m bray_curtis). Non-metric multidimensional scaling (NMDS), based on the Bray-Curtis distance, was used to visualize the composition of actinobacterial communities using the vegan package in R3.1.3 (R Core Team, 2012). We used an ANOSIM test to assess whether a significant variation of the environment was related to the actinobacterial composition or environment, to predict the metagenome function using script compare_categories.py in QIIME. The number of permutations was set at 999; all other arguments used the default values set. One-way PERMANOVA tests were used to test significant variation of actinobacterial community composition for each pair of environments with script compare_categories.py in QIIME. The number of permutations was set at 999. An assessment of the high-dimensional biomarker discovery and an explanation of the actinobacterial communities and metagenome gene functional content were performed using the LEfSe analysis (Segata et al., 2011). We used network analyses to explore co-occurrence patterns of actinobacterial communities for each environment. We first calculated the Pearson correlation coefficient of actinobacterial communities at the taxonomic level from species to class. Gephi software (version 0.9.2) was then used to visualize strongly correlated data with a *p* value < 0.01 and *r* > 0.6 or *r* < −0.6. The network layout algorithm used was the Fruchterman-Reingold Algorithm.

## Results

### Community Composition of Actinobacteria in Soils of the Heihe River Basin

**FIGURE 1:**
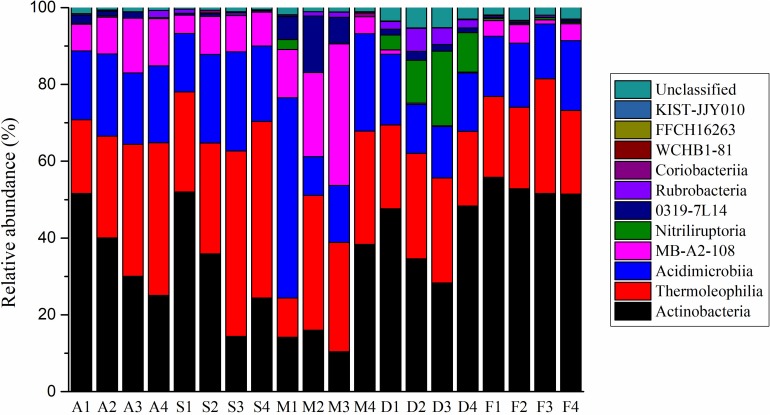
The actinobacterial community composition at the class level for the 20 sample sites.

A total of 110,773 (average 5 539 sample^–1^) actinobacterial sequences from the 20 soil sites were obtained from a total of 362 554 sequences. When grouped at the level of 97% similarity, 17,714 actinobacterial OTUs were formed. 12,010 OTUs contained only one sequence. The most abundant 20 OTUs were represented by 22,440 sequences or 20.3% of the total. The top 100 and 1,000 OTUs were 32.2% and 59.1%, respectively, of the entire data set. Ninety-seven percent of the actinobacterial sequences could be grouped into 11 classes (Figure 1 and Supplementary Figure S2). The mean relative abundances for each class were Actinobacteria (36.1%), Thermoleophilia (28.1%), Acidimicrobiia (19.4%), MB-A2-108 (8.4%), Nitriliruptoria (2.5%), 0319-7L14 (2.1%), Rubrobacteria (1.2%), Coriobacteriia (0.14%), WCHB1-81 (0.02%), FFCH16263 (0.006%) and KIST-JJY010 (0.005%). Only one class on the SILVA database, RBG-16-55-12, was not represented. At the lower level of classification, there were 33 orders, 83 families, 214 genera, and 247 species found in the Heihe river basin. *Streptomyces* genus sequences accounted for less than 1% of the total.

### Actinobacterial Communities of Habitat Generalists and Specialists

**FIGURE 2:**
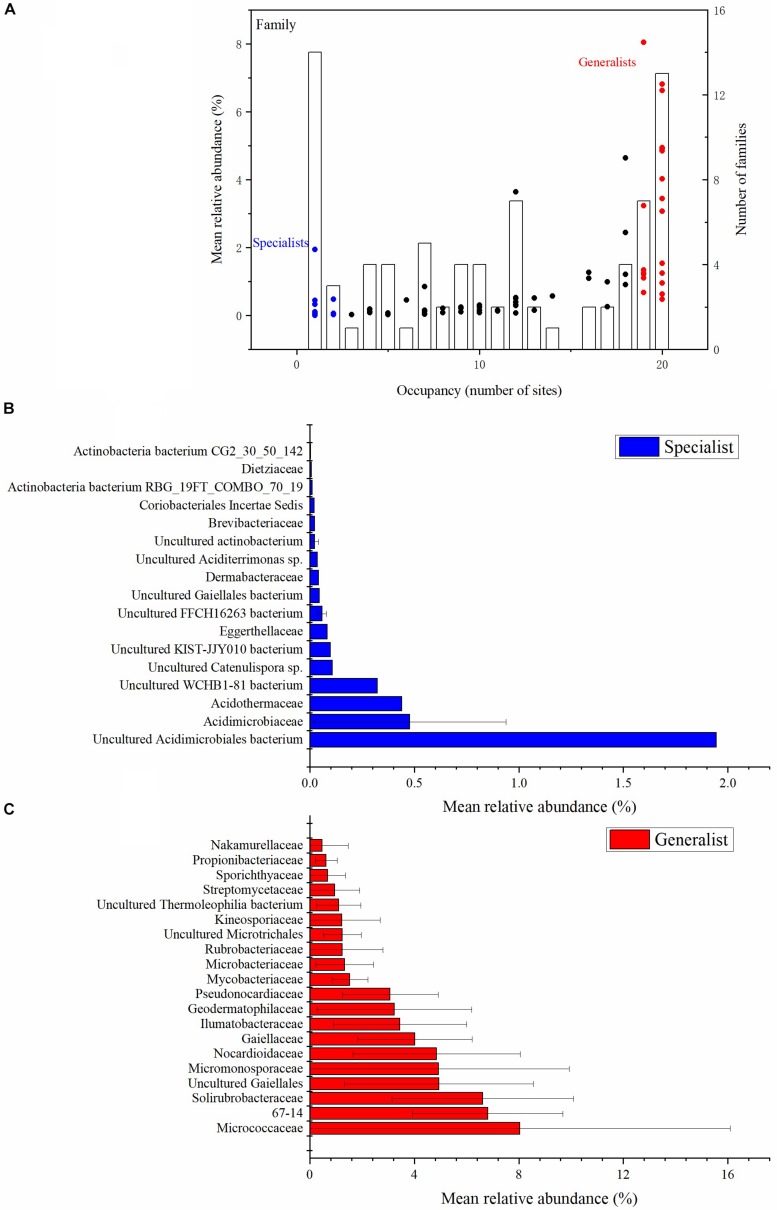
Abundance (*y* axis) and occupancy (*x* axis) plot for the actinobacterial communities at the family taxonomic level **(A)**. Occupancy indicates the number of sites that a family recovered. The mean relative abundance of families is indicated by dots (left-hand *y* axis). The specialist families are shown with blue dots. The generalist families are shown with red dots. The number of families at each occupancy is indicated by the bars (right-hand *y* axis) **(A)**. The mean relative abundance of the top 17 habitat specialist families **(B)** and top 20 habitat generalist families **(C)** are plotted with histograms (Unclassified sequences are not included). All the relative abundances in the figures are the proportion of the Actinobacteria phylum.

Each of the actinobacterial taxonomic units were assigned with the relative abundance vs. occupancy plot to split the set of taxa into two general categories: soil generalists and specialists (Figure 2 and Supplementary Figure S3). There were five class, 15 order, 20 family and 36 genus habitat generalists, and three class, seven order, 17 family and 69 genus habitat specialists. At the class taxonomic level, Actinobacteria, Thermoleophilia, Acidimicrobiia, MB-A2-108, and Rubrobacteria were the generalists, whereas WCHB1-81, FFCH16263, and KIST-JJY010 were the specialists. At the family taxonomic level, *Micrococcaceae*, *67-14*, and *Solirubrobacteraceae* were the main generalists, whereas *uncultured Acidimicrobiales bacteria*, *Acidimicrobiaceae*, and *Acidothermaceae* were the main specialists (Figure 2). At the genus taxonomic level, *Gaiella*, *Solirubrobacter*, *Nocardioides*, and *Blastococcus* were the main generalists, with *uncultured Acidimicrobiales bacterium*, *Collinsella*, and *uncultured prokaryote Gaiellales* being the main specialists (Supplementary Figure S3). The number of generalist genera was much higher than non-generalist genera (Supplementary Figure S3). More than half of the specialist genera belonged to the Actinobacteria class.

### Dissimilarity of Actinobacterial Communities Between Different Vegetation Ecosystems

In accordance with the taxonomic information from the SILVA database (Quast et al., 2013), the sequences could be grouped into unclassified, uncultured, and accurate classification. With a decrease of classification level, the ratio of unclassified and uncultured sequences increased (Supplementary Figure S2). The ratio of uncultured and accurate classified sequences from meadowlands were the highest of the five types of ecosystems. More differences appeared among unclassified, uncultured, and accurate classified sequences with a decrease in classification level across the five ecosystems.

**FIGURE 3:**
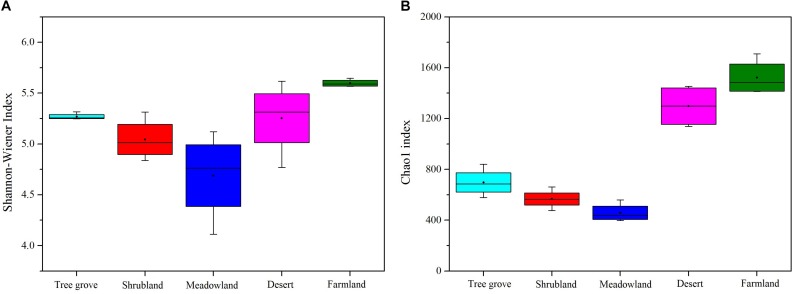
Boxplots showing Shannon-Wiener index **(A)** and Chao1 index **(B)** of actinobacterial communities in five biome types.

We used Chao1 and the ACE index to estimate the richness of actinobacterial communities, and the Shannon-Wiener and Simpson index to estimate the diversity of actinobacterial communities in the five different ecosystems (Figure 3 and Supplementary Figure S4). The results indicated that both richness and diversity of the actinobacterial communities followed the same trend. The highest was for the farmland ecosystem, followed by desert, tree grove, shrub, and meadow ecosystems. The diversity of samples belonging to the meadow ecosystem exhibited a greater variation range than for the other ecosystems, likely reflecting an association between community structure and plant species, as each of the four samples were obtained from soils adjacent to different plant species. The diversity in samples obtained from farmland and tree grove ecosystems was narrower than for the other ecosystems.

**FIGURE 4:**
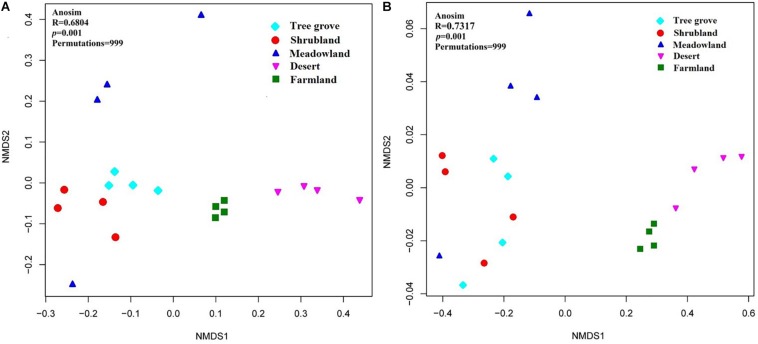
Non-metric multidimensional scaling (NMDS) biplot of a Bray-Curtis dissimilarity matrix that shows the actinobacterial community structure **(A)** and the functional metagenomic analysis **(B)** of the 20 sample sites.

Dissimilarities in the actinobacterial communities from the 20 sites were identified with a NMDS biplot (Figure 4). All sites, except M4, were aggregated with their environment. Notably, the composition of the actinobacterial communities was significantly affected by the environment (ANOSIM test, *R* = 0.6804, *p* = 0.001). The effect of five kinds of ecosystems on the actinobacterial communities was different (Supplementary Table S1). The four samples within the farmland environment shared the greatest similarity with a value of 0.82 ± 0.03. In contrast, the four samples from the meadowland had the greatest dissimilarity, with a value of 0.45 ± 0.18. Comparing the similarity of actinobacterial communities between each pair of environments, indicated that shrubland and tree grove ecosystems were the most similar environments (0.67 ± 0.09), whereas shrubland compared with either farmland or desert ecosystems exhibited the largest dissimilarity (0.36 ± 0.03, 0.36 ± 0.05). In addition, we used one-way PERMANOVA tests (Bray-Curtis distance and Euclidean distance) to analyze differences in actinobacterial communities for each environment pair, indicating that tree grove and shrubland communities were not significantly different. Pairwise comparisons of tree grove and meadowland communities, and shrubland and meadowland communities indicated marginal significant differences (Supplementary Tables S2, S3).

At the taxonomic class level, the Actinobacteria class were dominant in tree grove, desert and farm environments; Thermoleophilia was the largest class in shrub and meadow environments (Figure 1). At the level of family, there was more dissimilarity between the ecosystems (Supplementary Figure S5). Using LefSe analysis, we obtained actinobacterial biomarkers from class to species for each environment (Supplementary Figure S5). There were 29 biomarkers identified in tree grove samples, 22 in shrubland samples, two in meadowland samples, 45 in desert samples and 41 in farmland samples.

### Dissimilarity of Potential Function of Actinobacterial Communities

We categorized the KEGG results from metagenome analysis into hierarchies of functional pathways. Although there were different actinobacterial communities in the different habitats, the functional predicted pathways in the Actinobacteria from these different habitats were similar. The top five common metabolic predicted pathways were purine metabolism, butanoate metabolism, valine, leucine, and isoleucine degradation, propanoate metabolism and fatty acid metabolism. However, the top two most abundant specific functions were the Transporters and ABC transporters. They belong to the category Environmental Information Processing and took up to about 11% of all counts of the gene.

The dissimilarities in the potential functions (KO composition) of the actinobacterial communities among the 20 sites were identified with a NMDS biplot (Figure 4). Just as for the dissimilarity of actinobacterial communities, all sites, except M4, were aggregated with their environment. The metabolic potential function of the actinobacterial communities was significantly affected by the environment (Anosim test, *R* = 0.7317, *p* = 0.001). The analysis underlined that the different actinobacterial communities have different metabolic functions related to the ecosystem that they occupy.

In addition, LefSe analysis also indicated significant differences in potential metabolic functions in each environment (Supplementary Figure S6). This analysis identified 20 biomarkers for shrubland, 15 in meadowland, nine in farmland, 16 in desert and three in tree-grove soil.

**FIGURE 5:**
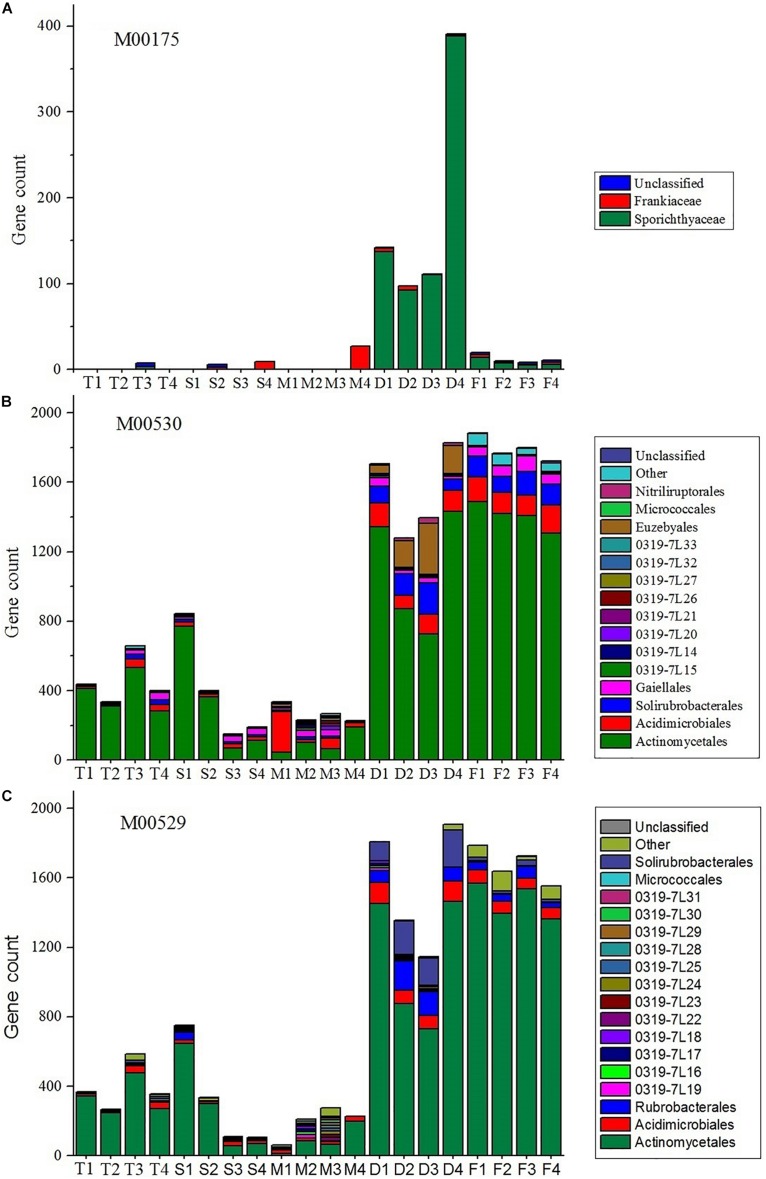
Stacked bar plots showing the estimated gene count contributions (*y*-axis) of the orders (≥2%) for M00175 (**A**, nitrogen fixation), M00530 (**B**, dissimilatory nitrate reduction), and M00529 (**C**, denitrification).

It is evident that nitrogen metabolism is an important feature of the actinobacterial communities, especially in farmland and non-cultivated desert ecosystems (Figure 5). *Sporichthyaceae*, abundant in the desert environment, were the main source of nitrogen fixation pathways (module M00175). The *Frankiaceae*, present in S4 and M4 samples, can also fix nitrogen. The dissimilatory nitrate reduction and denitrification pathways (modules M00530 and M00529, respectively), were also abundant in desert and farm ecosystems.

## Discussion

### Ecosystems That Vary in Vegetation Significantly Affect Actinobacterial Community Composition

Our analyses indicate that soil vegetation systems significantly influence the composition of actinobacterial communities in the Heihe river basin. The type of vegetation ecosystem plays a more important role in actinobacterial communities than the component plant species. This phenomenon is consistent with previous observations for how bacterial communities are affected by the type of ecosystem (Fierer et al., 2012b; Zhang et al., 2013; Xu et al., 2014). However, the influence of each of the different vegetation ecosystems on soil actinobacterial communities was different. For example, in this study, meadowland soil was the least rich and least diverse ecosystem (Figure 3) but contained non-clustering community structures (Figure 4), likely reflecting that actinobacterial communities vary according to the vegetation type. These results differ to the bacterial community analysis of Xu et al. (2014). They found that sample sites belonging to grasslands were intensively clustered together based on the bacterial community Bray-Curtis similarity scores. This suggests differences for how actinobacterial and overall bacteria communities can be influenced in the same kind of environment.

A previous analysis has indicated that the soil bacterial community structure is dependent on the specific plant species growing in the sample sites (Rich et al., 2003; Chu et al., 2011; Zhang et al., 2013; Xu et al., 2014). Our analyses of the Heihe river basin soils indicate that the actinobacterial communities in tree grove and shrubland soils are closely related, while they are distinct for three meadowland soil samples (Figure 4A). The exception was for site M4 that clustered with tree grove and shrubland soils. The soil from this site is associated with the plant *Achnatherum splendens* that may provide a soil micro-environment similar to the tree grove and shrubland soils. A functional metagenomic analysis also indicated a relation between the community structure of this site and that of tree grove and shrubland soils (Figure 4B). In these ecosystems, amino acid and carbohydrate metabolism pathways were better represented than in other sites. Different biomes typically harbor distinct plant species (Fierer et al., 2012b). Due to the diverse types of litter and rooting systems, the soil organic materials associated with different vegetation can vary (Chan et al., 2008; Zeng et al., 2016). This can consequently influence the soil microbial community structure.

### The Desert Ecosystem of the Heihe River Basin Is a Rich Resource for Actinobacteria

The desert ecosystem of the Heihe river basin is characterized as an abundant resource for Actinobacteria. It has a high alpha diversity index for uncultivated land (Shannon, Chao1, ACE) (Figure 3 and Supplementary Figure S4), has the lowest relative abundance of accurate classification sequences (Supplementary Figure S2), has the largest number of biomarker species and the lowest modularity index. The actinobacterial community in this desert ecosystem was different to those characterized in other desert ecosystems. Whereas the genera *Micromonospora*, *Streptosporangium* and *Cellulomonas* were found in desert soils from the northeast Qinghai-Tibet Plateau (Ding et al., 2013), they were not detected in the Heihe desert ecosystem. The genera *Microbispora* and *Microtetraspora* were found in soils from the Mojave Desert, California (Takahashi et al., 1996), but not in the desert soil of this study. In addition, the genus *Lechevalieria* was identified in soils from the Atacama Desert, Chile (Okoro et al., 2009), but was absent in this study. *Geodermatophilaceae* was the dominant group in both the Badain Jaran (27.4%) and Tengger (18.2%) deserts, but only 6.8% in this study (Sun et al., 2018). This suggests that the Actinobacteria communities in the Heihe desert are different from other deserts. Indeed, a variety of rare actinomycetes have been isolated from desert soils in the past few years (Tiwari and Gupta, 2013). More than two thirds of the rare actinomycete genera identified between 2006 and 2018, that are known to produce new natural products (Zhang et al., 2019), were present in the Heihe river basin desert soil. In contrast, the relative abundance of species belonging to the genus *Streptomyces* was no more than 1%.

### Different Actinobacterial Communities Inhabit Copiotrophic and Oligotrophic Ecosystems

The distribution of Actinobacteria has been associated with different environmental factors such as soil type and pH (Hayakawa et al., 1988; Matsukawa et al., 2007; Hayakawa, 2008). In many studies the abundance of actinobacterial communities is positively correlated with soil carbon and nitrogen content and are consequently considered copiotrophic bacteria. For example, the relative abundance of many dominant Actinobacteria was observed to increase with N gradients in agricultural fields (Fierer et al., 2012a). In addition, the abundance of Actinobacteria was positively correlated with soil carbon and nitrogen in tropical rain forest soils (Nemergut et al., 2010). Moreover, in nine different ecosystems examined in North America, nitrogen addition consistently led to an increase in soil Actinobacteria (Ramirez et al., 2012). Eilers et al. (2010) found that low molecular weight carbon compounds had significant effects on relative increases in Actinobacteria. However, we found that the relative abundance of Actinobacteria was higher in the oligotrophic environments (desert soil) and its community structure was significantly different compared to copiotrophic environments. For example, the class of Rubrobacteria and Nitriliruptoria were enriched in the desert environment. This suggests that some members of the phylum Actinobacteria may be able to live in an oligotrophic environment. Arocha-Garza et al. (2017) isolated 350 different strains from an oligotrophic desert oasis. The Actinobacteria can produce a variety of extracellular hydrolytic enzymes to degrade animal and plant residues, litter and other organic compounds in soil, enabling them to thrive in oligotrophic environments (Eisenlord and Zak, 2010). Shange et al. (2012) found that the orders of Actinomycetales and Solirubrobacterales showed their highest abundance in the lowest soil organic carbon values in a diverse agroecosystem. The genus *Saccharomonospora* was identified as the most abundant in relatively humus-poor alkaline soils (Hayakawa, 2008).

### Actinobacterial Communities May Play an Important Role in the Nitrogen Cycle in the Oligotrophic Desert Ecosystem

It is believed that actinobacterial communities play an important role in the ecosystems they colonize (Goodfellow and Williams, 1983; Holmalahti et al., 1994; Rich et al., 2003; Hill et al., 2011), although a functional description of their contribution is often lacking. A bioinformatic metagenome analysis provided us with insight into the potential function of Actinobacteria in the Heihe river basin ecosystems, suggesting an important role in nitrogen cycling in the oligotrophic desert ecosystem. In this environment, *Sporichthyaceae* and *Frankiaceae* would appear to be important for nitrogen fixation and may consequently enable other organisms to become established. In addition, members of the actinomycetales contribute significantly to nitrogen cycling in this environment. Previously, Navarrete et al. (2015) identified a close relationship between actinobacterial communities and nitrogen metabolism in copiotrophic Amazon forest soils. Our analysis indicates a potential role in nitrogen cycling for Actinobacteria as well as in the fertilized copiotrophic farmland ecosystem.

## Conclusion

This detailed description of actinobacterial communities in five different ecosystems of the Heihe river basin indicates that community structures vary according to the dominant vegetation and trophic status of the soils. This provides the data point to a surprisingly diverse community structure in oligotrophic desert soil, where actinobacteria could play an important role in nitrogen fixation. This habitat is also remarkable for its abundance of rare actinobacteria that are likely sources of new bioactive compounds.

## Data Availability Statement

The datasets generated for this study can be found in NCBI, KCVG00000000.

## Author Contributions

BZ, TC, and GL designed the study. XW, XT, and MW performed the field observation and sampling activities. BZ, LS, and XC analyzed the Illumina sequencing data. BZ, GZ, and WZ performed the statistical analysis. BZ, TC, GL, and PD interpreted the results and wrote the manuscript.

## Conflict of Interest

The authors declare that the research was conducted in the absence of any commercial or financial relationships that could be construed as a potential conflict of interest.
