# Supplementary material for: Variation in Actinobacterial Community Composition and Potential Function in Different Soil Ecosystems Belonging to the Arid Heihe River Basin of Northwest China
Source: Front Microbiol. 2019 Sep 24;10:2209. doi: 10.3389/fmicb.2019.02209 (PMC6769177; doi:10.3389/fmicb.2019.02209)
Supplement: Supplementary file 1 [file Data_Sheet_1.docx]

Table S1 Community structure similarity of actinobacteria within the same environment or between by pair-wise comparison

| Biome type | Tree grove | Shrubland | Meadowland | Desert | Farmland |
| --- | --- | --- | --- | --- | --- |
| Tree grove | 0.70±0.06 | 0.67±0.09 | 0.52±0.07 | 0.43±0.06 | 0.56±0.04 |
| Shrubland |  | 0.68±0.09 | 0.49±0.10 | 0.36±0.05 | 0.36±0.03 |
| Meadowland |  |  | 0.45±0.18 | 0.37±0.04 | 0.43±0.03 |
| Desert |  |  |  | 0.73±0.05 | 0.55±0.07 |
| Farmland |  |  |  |  | 0.82±0.03 |

Mean and standard deviation of Bray-Curtis similarities of all pairwise samples between any pair of environment were shown herein.

Table S2 Results of PERMANOVA tests comparing pair-wise actinobacterial community similarities derived in the matrix (Bray-Curtis) for each environment

| Biome type | Shrubland | | Meadowland | | Farmland | | Desert | |
| --- | --- | --- | --- | --- | --- | --- | --- | --- |
|  | F | *p* | F | *p* | F | *p* | F | *p* |
| Tree grove | 1.184 | 0.2309 | 1.859 | 0.0565 | 4.224 | 0.0352 | 5.675 | 0.0278 |
| Shrubland |  |  | 1.925 | 0.0838 | 4.730 | 0.0306 | 5.616 | 0.0300 |
| Meadowland |  |  |  |  | 2.999 | 0.029 | 3.280 | 0.0300 |
| Farmland |  |  |  |  |  |  | 5.058 | 0.0288 |

Table S3 Results of PERMANOVA tests comparing actinobacterial community similarity matrices (Euclidean distance) for any pair of environments

| Biome type | Shrubland | | Meadowland | | Farmland | | Desert | |
| --- | --- | --- | --- | --- | --- | --- | --- | --- |
|  | F | *p* | F | *p* | F | *p* | F | *p* |
| Tree grove | 0.6154 | 0.5463 | 1.691 | 0.0249 | 10.05 | 0.0278 | 4.483 | 0.0278 |
| Shrubland |  |  | 1.61 | 0.0597 | 9.78 | 0.0272 | 4.492 | 0.032 |
| Meadowland |  |  |  |  | 3.641 | 0.0291 | 4.316 | 0.0275 |
| Farmland |  |  |  |  |  |  | 4.194 | 0.0284 |


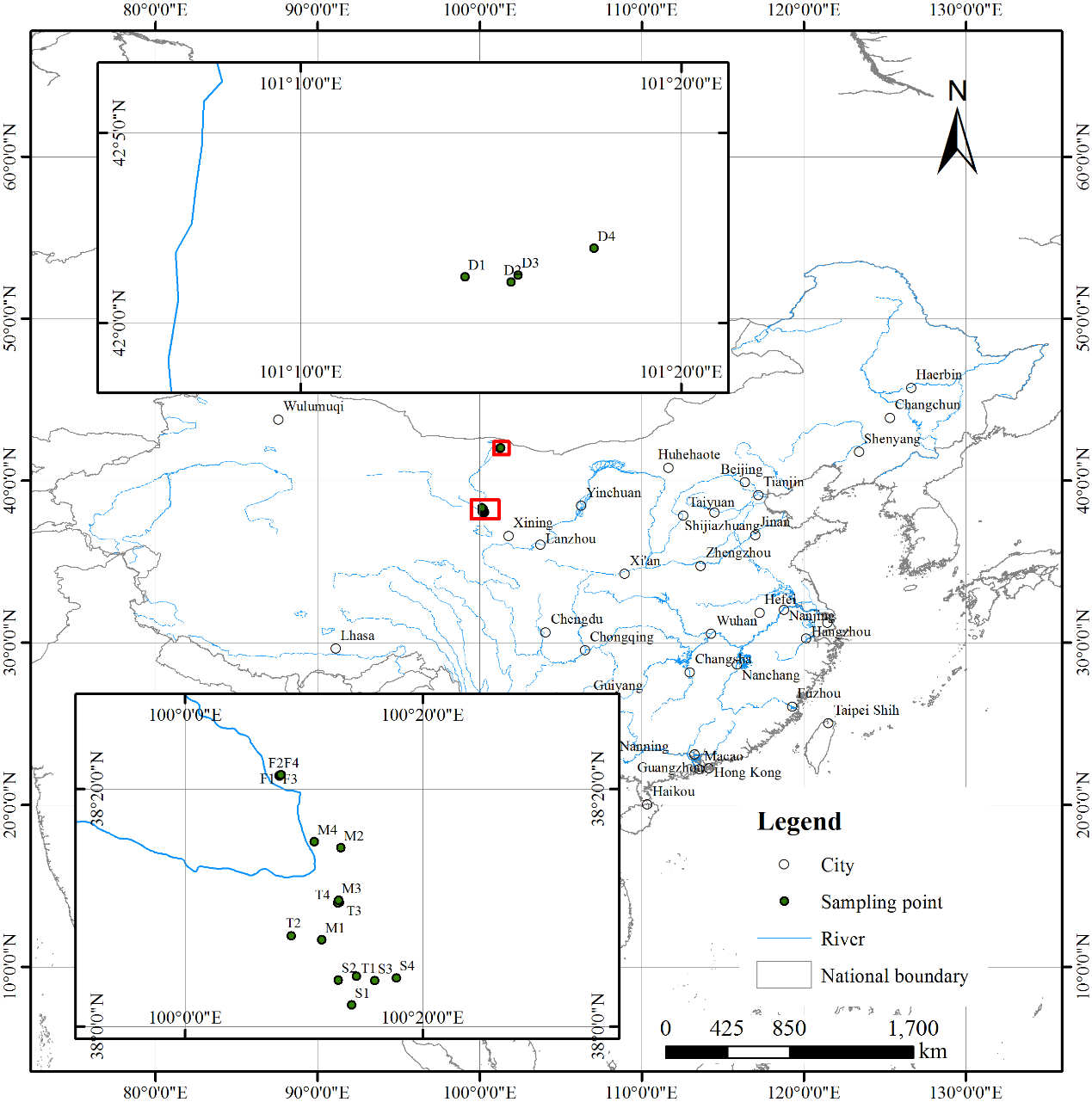
Figure S1 Map of sampling position


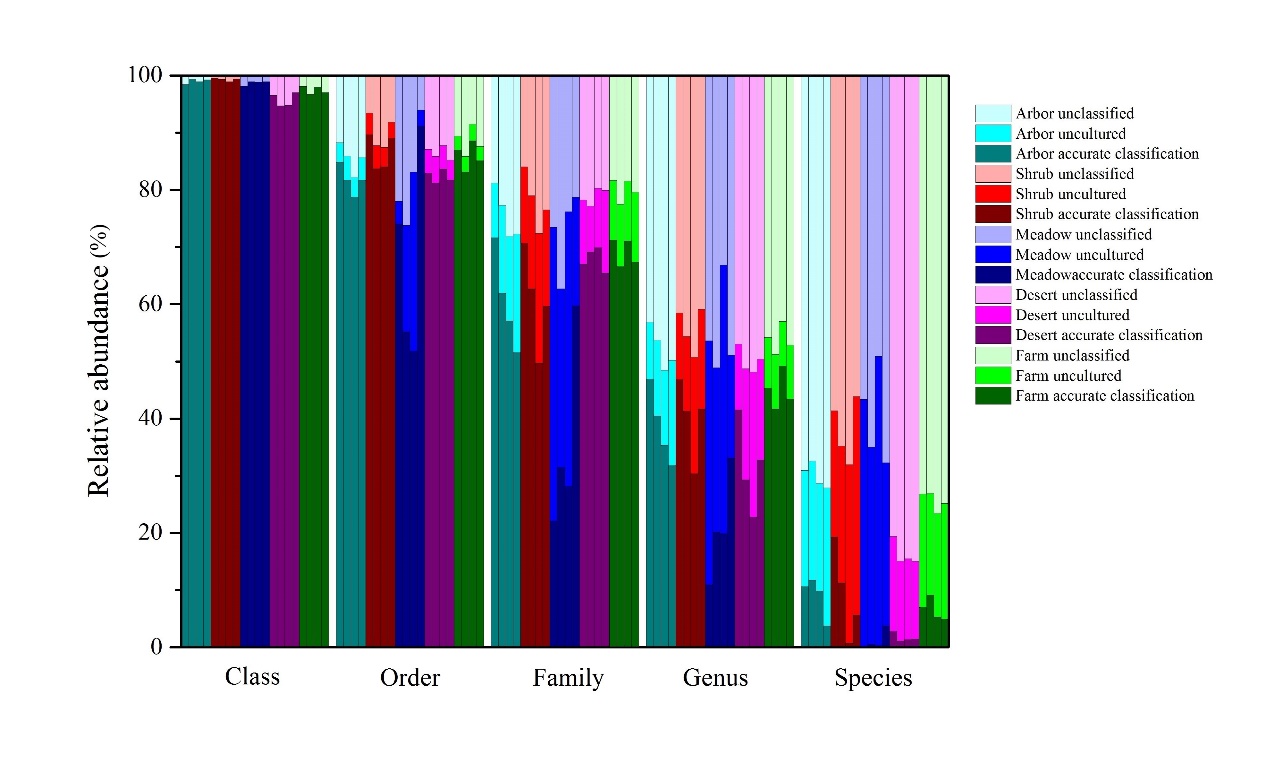


Figure S2 The relative abundance of unclassified, uncultured and accurate classified sequences in 20 samples represented at five taxonomic levels


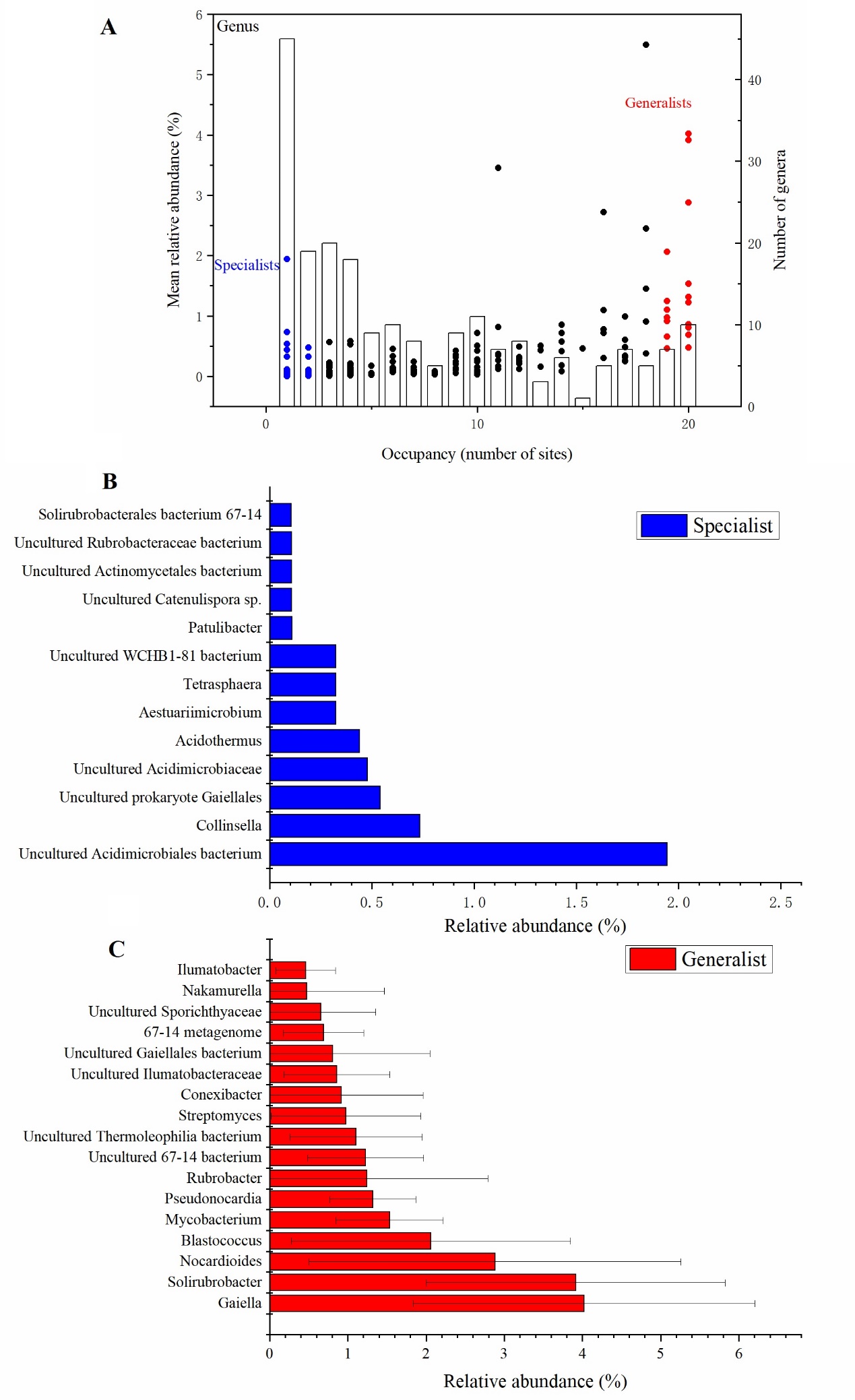


Figure S3 Abundance (y axis) and occupancy (x axis) plot for the actinobacterial communities at genus taxonomic level (A). Occupancy indicates the number of sites that a genus is recovered. The mean relative abundance of genera is indicated by dots (left-hand y axis). The specialist families are showed with blue dots. The generalist families are showed with red dots. The number of families at each occupancy is indicated by the bars (right-hand y axis) The number of shared genera in 1-20 samples is indicated with a broken line (A). The mean relative abundance of the top 13 habitat specialist genera (B) and top 17 habitat generalist genera (C) are plotted with histograms (Unclassified sequences are not included). All the relative abundances in the figures are the proportion of Actinobacteria phylum.


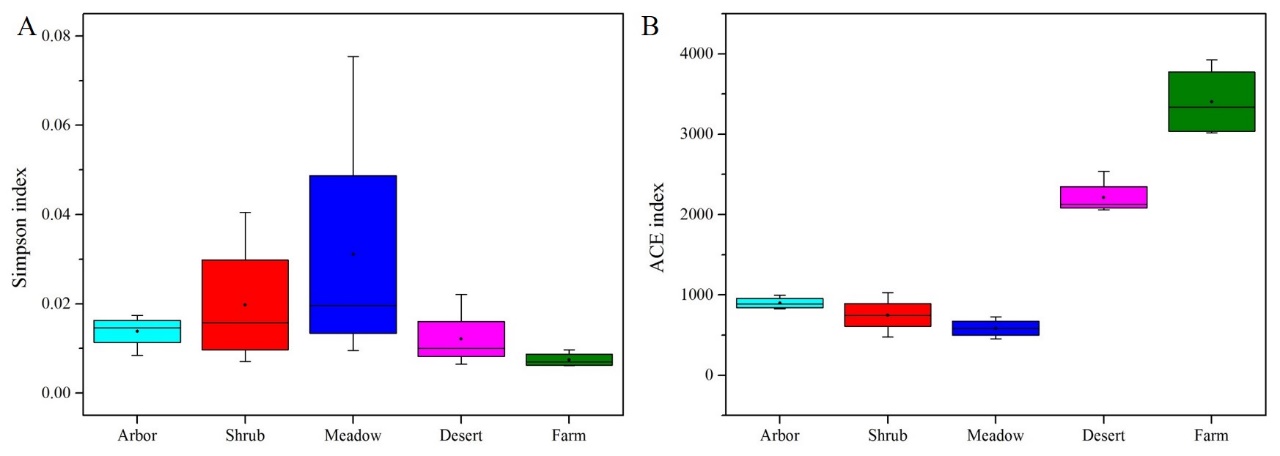


Figure S4 Boxplots showing Simpson index (A) and ACE index (B) of Actinobacterial communities in five biome types


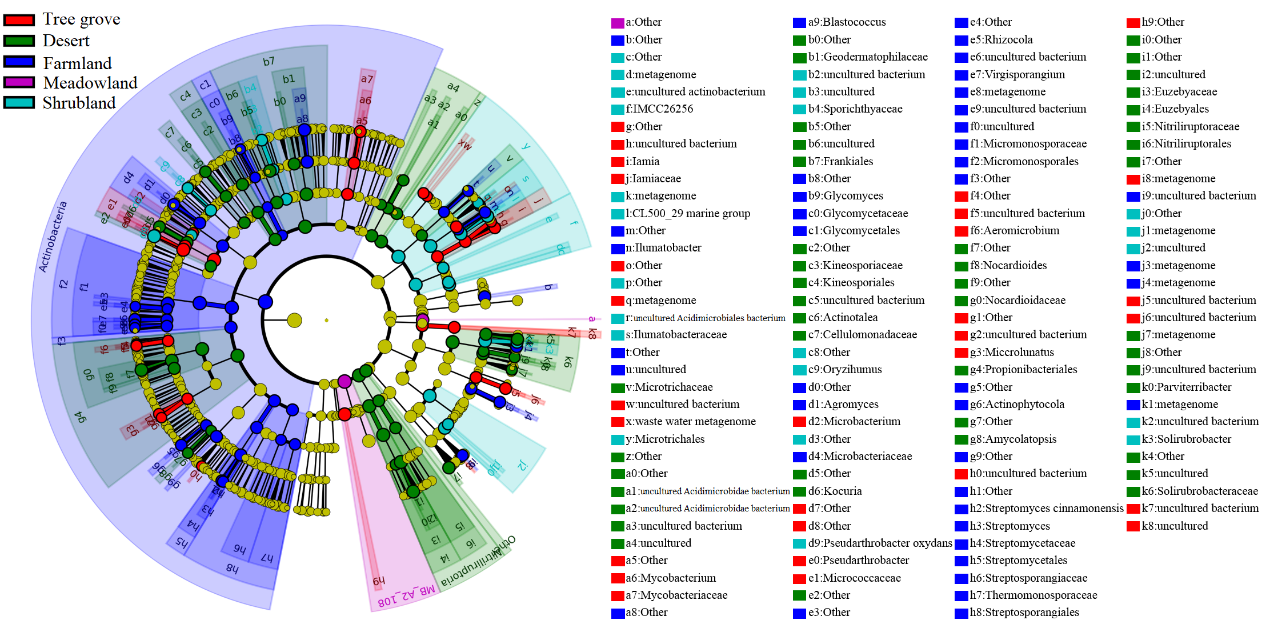


Figure S5 Taxonomic composition of actinobacterial community from five soil habitats.

In the evolutionary branch diagram, the circle radiating from inside to outside represents the classification level from the class (single circle) to the species. Each small circle at a different classification level represents a classification at that level, and the diameter of the small circle is proportional to the relative abundance. Colouring principle: Species without significant differences are uniformly coloured yellow, the differential species Biomarker follows the group for colouration, the red nodes represent the microbial groups that play an important role in the red group, and the green nodes indicate important roles in the green group. Other circles have similar meanings. The species names indicated by the English letters in the figure are shown in the legend on the right.


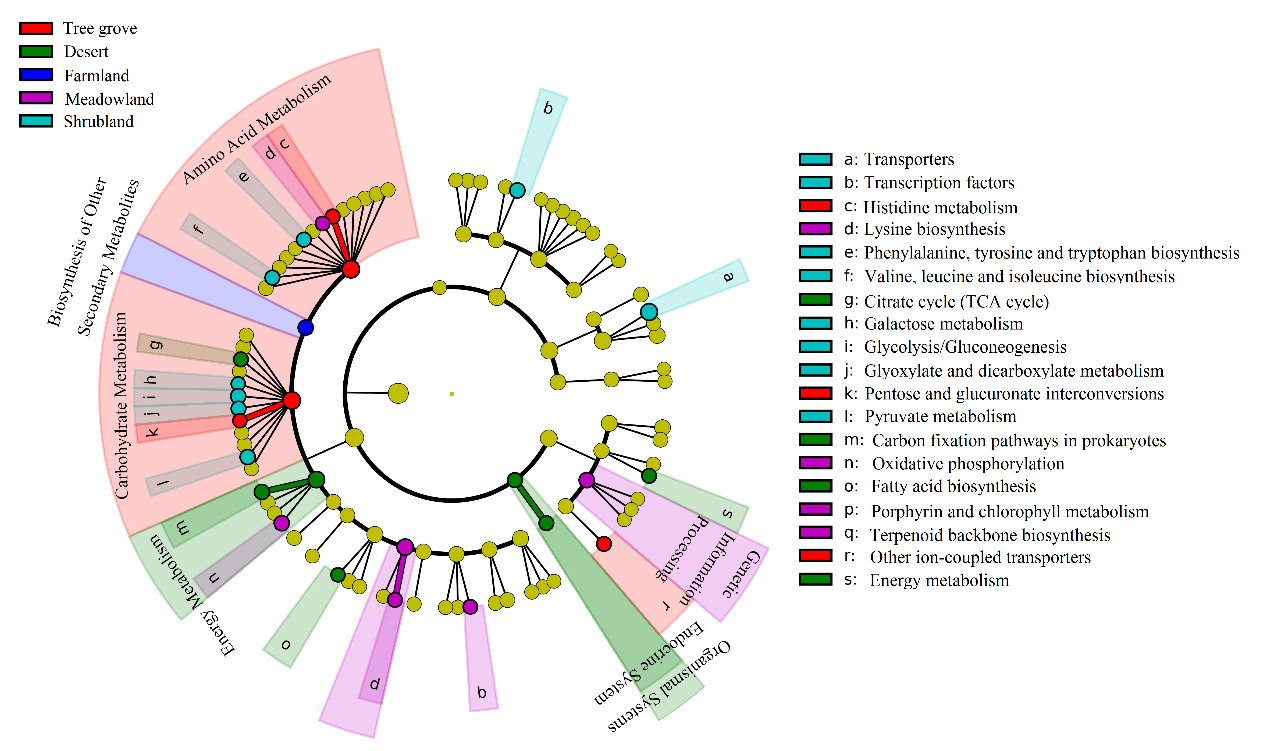


Figure S6 Taxonomic composition of predicted actinobacterial community metagenomic functions from five soil habitats.

In the evolutionary branch diagram, the circle radiating from inside to outside represents the KEGG_Pathways level. Each small circle at a different pathways level represents a pathways function at that level, and the diameter of the small circle is proportional to the relative abundance. Colouring principle: KEGG Pathways function without significant differences are uniformly coloured yellow, the differential KEGG Pathways function Biomarker follows the group for colouration, the red nodes represent the function groups that play an important role in the red group, and the green nodes indicate important roles in the green group. Other circles have similar meanings. The pathways function names indicated by the English letters in the figure are shown in the legend on the right.
